# Influence of Tariquidar, an ABC Transporter Inhibitor, on the Ca^2+^-Dependent Mitochondrial Permeability Transition Pore

**DOI:** 10.3390/ph18060924

**Published:** 2025-06-19

**Authors:** Tatiana A. Fedotcheva, Alexey G. Kruglov, Nadezhda I. Fedotcheva

**Affiliations:** 1 Science Research Laboratory of Molecular Pharmacology, Medical Biological Faculty, Pirogov Russian National Research Medical University, Ministry of Health of the Russian Federation, 1, Ostrovityanova st., Moscow 117997, Russia; tfedotcheva@mail.ru; 2 Institute of Theoretical and Experimental Biophysics, Russian Academy of Sciences, 3, Institutskaya st., Pushchino 142290, Russia; krugalex@rambler.ru

**Keywords:** tariquidar, multidrug resistance, mitochondria, mitochondrial permeability transition pore, adenine nucleotide translocase

## Abstract

**Background**: Tariquidar (Tq) is an inhibitor of the multidrug resistance (MDR) proteins relevant to ATP-binding cassette transporters (ABC transporters), which suppresses the ATP-dependent efflux of a variety of hydrophilic and amphipathic compounds, including anticancer drugs. Tq is a representative of a new generation of MDR inhibitors with high affinity to ABC proteins. However, there are still no data on the possible effect of Tq on mitochondria as an important target in the regulation of cell death or survival. **Methods**: We investigated the influence of Tq on the Ca^2+^-dependent mitochondrial permeability transition pore (mPTP). The effect of Tq was assessed using several parameters, including the calcium load, membrane potential, and mitochondrial swelling. To evaluate the specific targets of Tq, selective inhibitors of components of the mitochondrial pore were used, including adenine nucleotides, carboxyatractylozide (Catr) and bongkrekic acid (BA), oligomycin, and cyclosporine A. **Results**: Tq decreased the calcium retention capacity, activated mitochondrial swelling, and lowered the influence of ADP and ATP, the inhibitors of the Ca^2+^-induced pore opening, at their low concentrations. These effects of Tq were observed in both calcium-load and swelling assays, thus mimicking the effect of Catr, a selective inhibitor of adenine nucleotide translocase (ANT). Tq also decreased the protective effect of BA, an inhibitor of ANT and mPTP, on the calcium retention capacity of mitochondria. Further, Tq dose-dependently decreased the inhibitory effect of a low ATP concentration but not of high concentrations, at which the effect of Tq was activated by oligomycin, an inhibitor of F-ATP synthase. **Conclusions**: The influence of Tq extends to mitochondria, specifically to the regulation of membrane permeability, promoting the activation of pore opening, probably through an interaction with ANT, a component of the pore-forming complex. The effect of Tq on the opening of mPTP is strongly dependent on the concentrations of adenine nucleotides and, consequently, on the functional state of mitochondria. The direct influence of Tq on mitochondria can be considered as a new activity that promotes the sensitization of cells to various treatments and stimuli.

## 1. Introduction

Tariquidar (Tq) is an inhibitor of multidrug resistance (MDR) proteins related to ATP-binding cassette transporters (ABC transporters). ABC transporters provide the ATP-dependent efflux of low-molecular compounds, including drugs, across plasma membranes. These transporters have two cytoplasmic nucleotide-binding domains (NBDs), which hydrolyze ATP, and two transmembrane domains (TMDs), which bind substrate molecules and promote their translocation across a membrane [1,2,3]. Their inhibition enhances intracellular drug accumulation and retention [4,5,6]. It is believed that there are three ways to reduce chemotherapy resistance: inhibiting the activity of the transporter, inhibiting the expression of P-glycoprotein, and silencing or knocking out the related gene [7]. The inhibitors of the first of these pathways target the drug-binding domain or the nucleotide-binding domain responsible for ATP binding and hydrolysis [8].

**Scheme 1:**
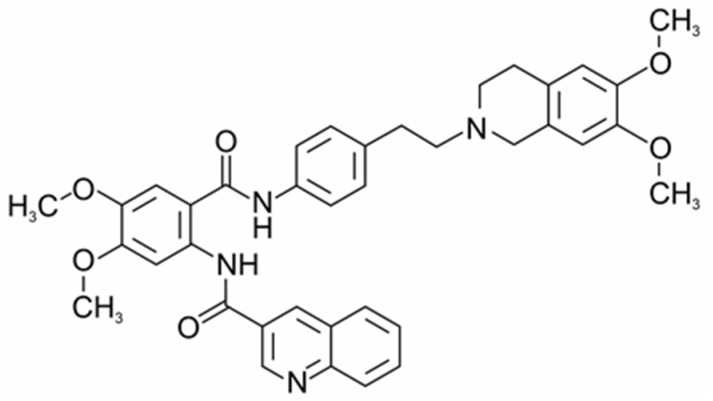
Chemical structure of tariquidar (Inxight Drugs, https://drugs.ncats.io/substance/J58862DTVD, accessed on 8 June 2025) [12].

Tq, a tetrahydroisoquinoline derivative (Scheme 1), belongs to a new generation of inhibitors of MDR proteins, the chemical structure of which determines their ability to directly bind to drug-binding sites due to hydrophobic interactions with proteins, as well as to block ATP binding and hydrolysis [9,10]. As has been shown, Tq inhibits ATP hydrolysis and the transport function coupled with ATP hydrolysis [11]. The high hydrophobicity of the compound suggests its preferential distribution in lipid membranes [6,9,10]

In preclinical and clinical trials, Tq has demonstrated significant results as a subsidiary agent against drug-resistant cancers [13,14]. Recently, Tq has been shown to effectively inhibit an ABC transporter (P-gp) at the human blood–brain barrier, improving the entry of several antiseizure medications into the brain in drug-resistant epilepsy [15]. Tq significantly reduced the viability of medulloblastoma cells, increased the plasma and brain concentrations of a specific drug, and prolonged the drug plasma half-life by several times [16]. Also, the co-administration of Tq increased the exposure of the CNS to drugs in the treatment of neuropathic pain [17].

In addition to ABC transporter-dependent factors, chemoresistance may involve several cellular protection mechanisms, including mitochondria-related mechanisms such as metabolism, Ca^2+^ signaling, and the mitochondria-dependent apoptosis underlying cell survival or death [18,19]. There is evidence showing the simultaneous influence of some inhibitors on both MDR-related proteins and mitochondrial functions [20,21]. We have also shown that the actions of quinidine and verapamil, the first-generation inhibitors, extend to mitochondria, in particular, to nonspecific membrane permeability [22]. It was supposed that their effects are associated with the positive charges and amphipathic properties of these membrane-active drugs.

The drug-resistant cells of numerous cancer types perform the deregulation of mitochondrial functions, including increased biogenesis, as well as metabolic and energetic rearrangements; therefore, mitochondria-targeting therapeutic strategies might help reduce cancer progression and chemoresistance [23]. Mitochondria are involved in metabolic quality control and the survival of cells. It is known that the mitochondrial permeability transition pore (mPTP) opening is a critical event in the induction of apoptosis; consequently, targeting the mPTP components with the subsequent induction of apoptosis may bypass the chemoresistance [18,19].

Calcium ions are the most potent inducers of the mPTP. Mitochondrial Ca^2+^ signaling pathways are attracting growing interest since the proteins involved in these pathways may represent new targets for cancer therapy. Ca^2+^ buffering in mitochondria is pivotal for the maintenance of mitochondrial integrity, energy production, redox balance, and apoptosis [24]. Although mitochondria can accumulate large amounts of calcium ions without mPTP activation, the sensitivity of mitochondria to calcium loading can vary in response to different factors [25]. In the present work, we investigated the influence of Tq on the Ca^2+^-dependent mPTP opening. The effect of Tq was assessed using several parameters, including the calcium load, the membrane potential, and mitochondrial swelling. To evaluate the specific targets of Tq, selective inhibitors of the components of the mitochondrial pore were used, including adenine nucleotides, carboxyatractylozide (Catr) and bongkrekic acid (BA), oligomycin, and cyclosporine A.

## 2. Results

### 2.1. Influence of Tq on Resistance to Calcium Load and Ca^2+^-Induced mPTP Opening

**Figure 1:**
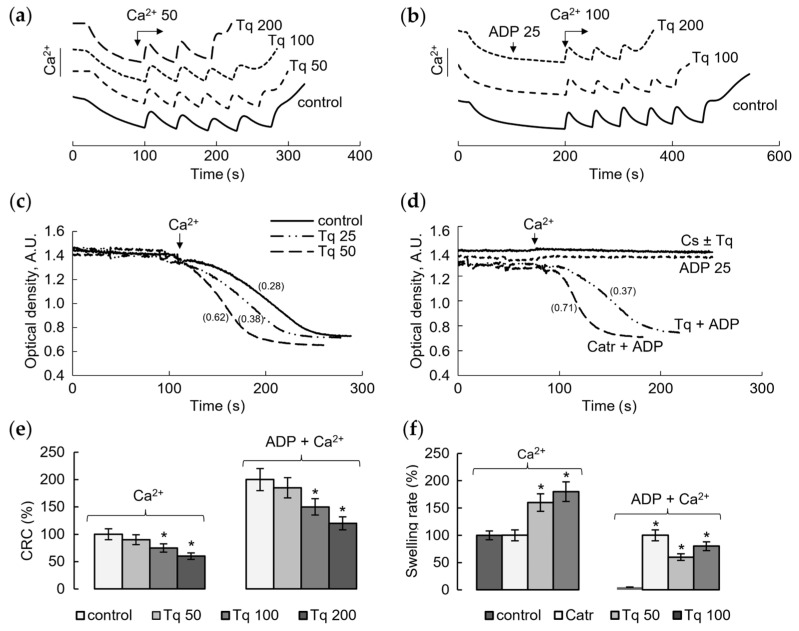
Influence of Tq on the Ca^2+^-induced mPTP opening. The calcium retention capacity (CRC) of mitochondria in the course of successive additions of 50 μM CaCl_2_ in the control (**a**) and upon supplementation of 25 μM ADP and additions of CaCl_2_, at a concentration of 100 μM each (**b**), in the presence of Tq at the indicated concentrations; the influence of Tq on the Ca^2+^-induced (50 μM) mitochondrial swelling in control mitochondria (**c**) and in the presence of mPTP inhibitors Cs A (1 µM), ADP (25 µM), and Catr (1 µM) (**d**); the influence of Tq at different concentrations on the calcium load (**e**) and the rate of mitochondrial swelling (**f**) in the control and in the presence of ADP. An asterisk (*) indicates the values ± S.D. that differ significantly from the control values (*p* < 0.05); the swelling rates (Δ540 nm/min) are indicated in parentheses; all measurements were carried out in a buffer containing 125 mM KCl, 15 mM HEPES, 1.5 mM phosphate, and 5 mM succinate.

We tested the influence of Tq on the calcium retention capacity and mitochondrial swelling, which are the main criteria for the mPTP opening by calcium ions. The changes in these parameters reflect the involvement of Tq in the regulation of the calcium-induced mPTP opening. Figure 1 shows the influence of Tq at different concentrations on the mPTP opening induced by calcium ions. It is seen that Tq decreased the threshold calcium ion concentrations required for the pore opening, which were determined in the course of successive calcium additions (Figure 1a). Tq began to act on this parameter at concentrations above 50 μM, reaching a 40% decrease at a concentration of 200 μM. The involvement of ANT in the effect of Tq was assessed by changes in the capacity to retain the maximum calcium concentrations in the matrix in the presence of ADP, an inhibitor of mPTP. As shown in Figure 1b, ADP at a low concentration (25 μM) increased the calcium retention capacity more than twofold. Under these conditions, Tq activated the mPTP opening, reducing the effect of ADP by 25% at a concentration of 100 μM and almost completely at a concentration of 200 μM. Thus, Tq activates the mPTP opening, diminishing the resistance to the calcium load and restricting the protective effect of ADP. The latter shows that a target of Tq action may be ANT, a component of the pore-forming complex.

In the next experiments, we tested the influence of Tq on mitochondrial swelling as another criterion of mPTP opening. As shown in Figure 1c, Tq increased the rate of calcium ion-induced mitochondrial swelling in a concentration-dependent manner. At a Tq concentration of 50–100 μM, the swelling rate increased twofold compared to the control. Figure 1d shows the effect of Tq on the mitochondrial swelling in the presence of cyclosporine A (CsA) and ADP, the inhibitors of mPTP. Tq did not abolish the inhibition of swelling in the presence of CsA (Figure 1d). However, it eliminated the inhibition caused by ADP at a low (25 μM) concentration. This effect of Tq is similar to the effect of Catr, a specific ANT inhibitor, which acts from the outside the inner mitochondrial membrane (Figure 1d). Thus, Tq sensitizes the opening of mPTP, decreasing the resistance to calcium loading and activating the swelling (Figure 1e,f). Both effects are enhanced by increasing the Tq concentration; changes in these parameters reach 50% at a concentration of about 100–200 μM.

### 2.2. Influence of Tq on the Membrane Potential and Resistance to the Calcium Load and ANT Inhibitors

**Figure 2:**
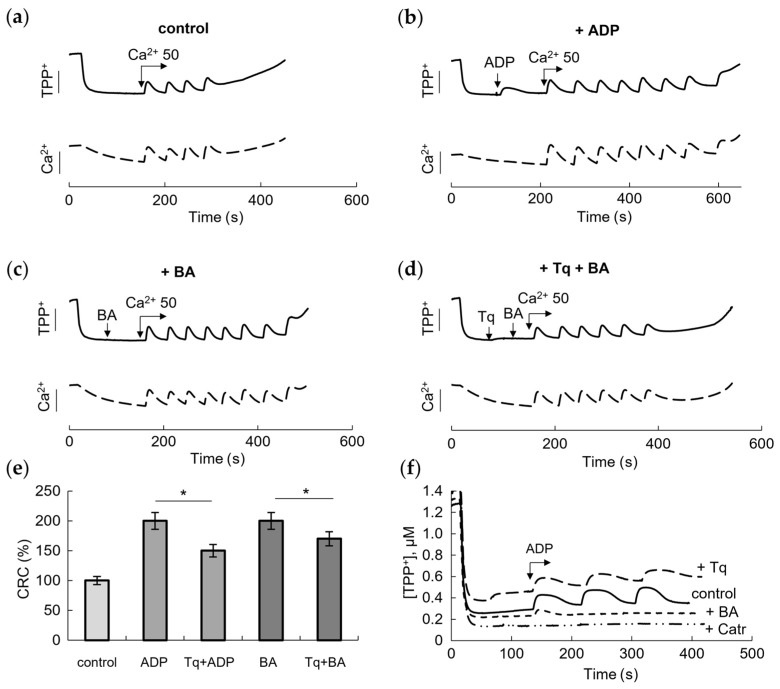
Influence of Tq and bongkrekic acid (BA) on the membrane potential and Ca^2+^-induced mPTP opening. Changes in the membrane potential and the calcium retention capacity (CRC) of mitochondria during successive additions of 50 μM CaCl_2_ in the control (**a**), upon supplementation of 25 μM ADP (**b**), 10 μM bongkrekic acid (BA) (**c**) and 100 μM Tq with BA (**d**). Comparison of the effects of Tq and inhibitors on the CRC (**e**) and ADP-induced decrease/restoration of the membrane potential (**f**). An asterisk (*) indicates the values ± S.D. that differ significantly from the control values (*p* < 0.05).

To verify the implication of ANT to the effect of Tq, we compared its influence with the effects of the ANT inhibitors BA and Catr on the mPTP opening. In these experiments, the effect of Tq and the inhibitors was assessed by changes in the membrane potential and the resistance to the calcium load with the simultaneous recording of TPP+ and calcium ion concentrations during successive calcium additions. As the pore opened, mitochondria lost the ability to restore the membrane potential and accumulate the next portion of calcium. Figure 2 shows these changes in the control (Figure 2a), in the presence of a low (25 μM) concentration of ADP (Figure 2b) and in the presence of 10 μM BA (Figure 2c).

As expected, BA inhibited pore opening by increasing the amount of added calcium nearly twofold, acting similarly to ADP. In the above experiment (Figure 1b,d), we showed that Tq partially abolished the protective effect of ADP. Since BA is a selective inhibitor of ANT, which acts from the side of the mitochondrial matrix, and also an inhibitor of mPTP opening, it was important to test the influence of Tq on the effect of BA. As shown in Figure 2d, Tq reduced the protective effect of BA by decreasing the CRC by two calcium supplements.

Summarizing the data presented in Figure 1 and Figure 2, it can be seen that Tq is more effective in relieving the inhibition by ADP than the inhibition by BA (Figure 2e). At a concentration of 100 μM, Tq reduced the effects of ADP and BA by about 50% and 25%, respectively. We also compared the effect of each inhibitor on the decrease in/recovery of the membrane potential during three successive additions of ADP at a concentration of 25 μM (Figure 2f). Changes in the membrane potential in response to the addition of ADP reflect, among other things, the participation of ANT in oxidative phosphorylation. The contribution of ANT is especially evident in the presence of Catr, which completely destroys the effect of ADP. BA also removed the effect of ADP on the membrane potential, showing a small shift only after the first addition of ADP. Tq did not remove the effect of ADP but significantly slowed down, by about 25%, the restoration of the membrane potential after each addition of ADP, which may also indicate a dysfunction of the carrier.

### 2.3. Modulation of the Ca^2+^-Induced mPTP Opening by Tq at Different Concentrations of Adenine Nucleotides

In the next experiments, we assessed the effect of Tq on the opening of mPTP during continuous incubation with mitochondria in the presence of subthreshold concentrations of calcium ions and different, including physiological, concentrations of ATP. The influence of oligomycin, an ATPase inhibitor, on the opening of mPTP in the presence of Tq was also examined.

**Figure 3:**
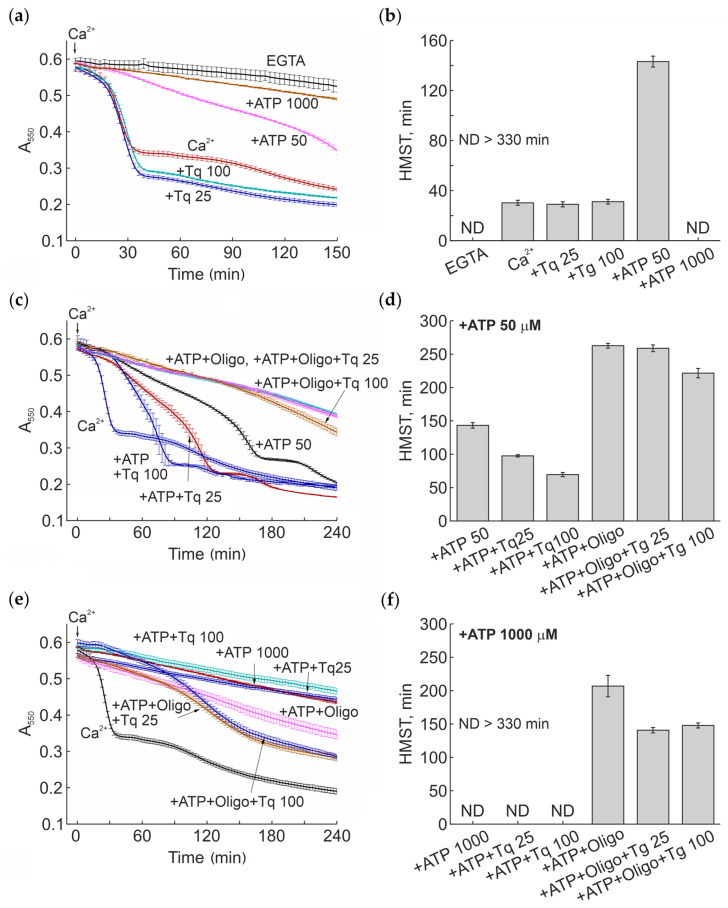
Effect of Tq on the mitochondrial Ca^2+^-dependent swelling (PTP opening) in the presence of different ATP concentrations and oligomycin. Mitochondria (0.75 mg prot./mL) were placed in incubation medium containing 5 mM glutamate, 5 mM malate, and 10 µM EGTA. Where indicated, the medium also contained 50 and 1000 µM ATP, 25 and 100 µM Tq, and 5 µM oligomycin (Oligo). The arrow shows the addition of 25 µM CaCl_2_. Panels (**a**), (**c**), and (**e**) show original traces of mitochondrial swelling of one standard experiment of three similar. Points of the curves are the means ± S.D. of the signal from three wells of a plate (technical replicates). On panels (**b**), (**d**), and (**f**) are the values of the half-maximum swelling time (HMST) (mean ± S.D.) for experiments in panels (**a**), (**c**), and (**e**), respectively. ND > 330 min, HMST was not determined since the half-maximum swelling was not observed during the time of experiment.

Figure 3 shows that, in the presence of Ca^2+^ at a low subthreshold concentration, Tq minimally affected the rate of mitochondrial swelling (half-maximum swelling time (HMST), i.e., the time when half the mitochondria become swollen due to PTP opening) (Figure 3a,b). Simultaneously, Tq increased the amplitude of swelling. As shown, ATP strongly and dose-dependently inhibited the swelling: 50 µM and 1 mM ATP increased the HMST by about 5-times and by more than 11-times, respectively. In the presence of 1 mM ATP, the half-maximum swelling was not observed during the experiments. In turn, Tq dose-dependently decreased the inhibitory effect of a low (50 µM) ATP concentration (by about two-times) (Figure 3c,d) but was unable to lower the effect of a high (1 mM) concentration (Figure 3e,f). Oligomycin, an inhibitor of F-ATP synthase, increased and decreased the protective effect of ATP at low and high concentrations, respectively (Figure 3c–f). In the presence of 1 mM ATP, Tq significantly accelerated the mitochondrial swelling induced by the presence of oligomycin. Under these conditions, Tq activated the swelling already at a concentration of 25 μM (Figure 3e,f). These data show that Tq stimulates the pore opening upon prolonged incubation in the presence of subthreshold concentrations of calcium ions and a low ATP concentration, while at physiological concentrations of ATP, the effect of Tq was not evident but could be activated by oligomycin.

## 3. Discussion

The permeability transition pores in mitochondria and ATP-binding cassette transporters in cell membranes are the systems of nonspecific membrane transport, which provide the efflux of low-molecular compounds across mitochondrial and plasma membranes, respectively. Both are of fundamental importance for cell survival in normal and pathological conditions. There is evidence indicating the simultaneous influence of inhibitors on both MDR proteins and mitochondrial functions. Our results show that the influence of Tq extends to mitochondria, manifesting itself in the stimulation of pore opening by calcium ions. Tq decreases the mitochondrial resistance to calcium stress and increases the rate of swelling induced by threshold calcium concentrations. The most significant effect is the elimination of the protective action of ADP and ATP against mPTP opening, which is similar to the effect of Catr, a selective ANT inhibitor. Also, as follows from our data, the effect of Tq on the opening of mPTP is strongly dependent on the concentration of adenine nucleotides.

ADP, ATP, BA, and Catr are specific regulators of the opening of the cyclosporin A-sensitive mPTP. As is known, Catr and calcium stabilize ANT in the cytosolic (“c”) conformation (the nucleotide-binding site facing the cytosol) and stimulate mPTP opening, whereas BA stabilizes ANT in the matrix (“m”) conformational state and inhibits mPTP opening. By contrast, adenine nucleotides suppress Ca^2+^-induced mPTP opening through the binding with ANT, while Catr interferes with adenine nucleotide binding and sensitizes the mPTP to calcium ions [26,27]. Presumably, Tq influences the mPTP opening similarly to Catr, relieving the ADP-mediated pore inhibition. According to our data, Tq decreased the protective effect of BA, an inhibitor of ANT and mPTP, on the calcium retention capacity of mitochondria, which also supports this assumption.

As previously reported, the conformational changes of ANT may correlate with drug resistance. For instance, Catr improved the chemosensitivity of cancer cells to cisplatin [28]. Recently, it has been shown that adenine nucleotide translocase 3 (ANT3) may be a therapeutic target in the multiple myeloma progression and drug resistance mediated by a complex mechanism of expression and traffic of regulatory proteins of mitophagy [29]. In addition, the overexpression and the knockdown of ANT isoforms modulate the sensitivity of cells to apoptotic stimuli in cases where different isoforms have opposite effects on cell survival [30,31]. ANT1 and ANT3 isoforms act as pro-apoptotic factors, while ANT2 and ANT4 isoforms provide the resistance to death-inducing stimuli [31,32,33]. Thus, differences in the expression of certain ANT isoforms may be responsible for the resistance of cancer cells to apoptosis. These data demonstrate the important role of ANT in maintaining the viability of tumor cells or the initiation of their death. Interestingly, similar changes in the conformation of drug-binding domains are thought to occur upon the binding and release of drugs by ABC transporters. A change from the inward-facing to the outward-facing conformation is considered to be triggered by the binding of ATP, whereas a change back to the inward-facing conformation is powered by the hydrolysis of ATP [8].

The tetrahydroisoquinoline derivative Tq is characterized by a high affinity for ABC proteins [9,34]. Biochemical investigations along with transport studies in intact cells showed that Tq binds at a high-affinity site to cause the inhibition of ATP hydrolysis and the transport function of some ABC transporters [11]. As follows from our data, the effect of Tq on the opening of mPTP is strongly dependent on the concentration of adenine nucleotides. The dependence of the influence of anti-MDR drugs on the functional state of mitochondria has been revealed in many studies and attributed to changes in the energy production, calcium homeostasis and signaling, as well as to metabolic remodeling in tumor cells [18,19]. The effect of oligomycin on the sensitization of the mPTP opening to Tq, found in our experiments, may be also associated with changes in the balance of adenine nucleotides and calcium. A similar effect of oligomycin, aimed at inducing the mPTP opening and activating the apoptosis, was observed in cancer-associated fibroblasts and explained by disrupted calcium homeostasis, since the influence of oligomycin was mitigated by calcium chelation [35]. It is also important to emphasize that the oligomycin sensitivity-conferring protein (OSCP), a component of F1Fo-ATP synthase in mitochondria, is closely related to the regulation of the mPTP due to the interaction with some regulatory proteins, including cyclophilin D. This interaction partially inhibits the enzymatic activity of F1Fo-ATP synthase and simultaneously increases the sensitivity of the mPTP to Ca^2+^ [36]. Our data show that the effects of oligomycin and Tq are dependent on the ATP concentration, which may indicate the involvement of both energetic and conformational changes in the observed activation of the mPTP. Both ANT and FoF1-ATPase play a critical role in ATP synthesis and the formation of the mitochondrial permeability transition pore complex, and both are considered as possible targets in antitumor therapy [37,38]. In addition, in the presence of oligomycin, the level of ATP decreases due to its consumption to maintain other functional parameters of mitochondria such as the membrane potential, ion homeostasis, and other relevant characteristics. It is believed that tumor cells primarily support these parameters for mitochondrial stability using ATP that is derived from glycolysis [39]. This mode of ATP consumption probably occurs in the presence of oligomycin in our experiments.

Although our data show that the effect of Tq is sufficiently specific to ANT, its influence as a large lipophilic molecule on membrane lipids and the protein conformation cannot be excluded. As has been recently shown, free Tq in the lipid membrane predominantly acquires extended conformations, which can improve the access to binding sites [40]. Also, as has been previously assumed, the incorporation of lipophilic molecules into the phospholipid membrane may contribute to changes in the conformation of the membrane-integrated proteins of the plasma membrane, including MDR proteins [41]. This property can be brought about in both plasma and mitochondrial membranes and underlies the effect of Tq on the adenine nucleotide-dependent regulation of the permeability of a mitochondrial membrane, facilitating the interaction with pore-forming proteins.

## 4. Materials and Methods

### 4.1. Reagents and Chemicals

Tariquidar dihydrochloride was obtained from TOCRIS (Tocris Bioscienes, Bristol, UK). All other reagents (adenosine diphosphate, carboxyatractylozide, bongkrekic acid, oligomycin, cyclosporine A, and others) were from the Sigma–Aldrich Corporation (St. Louis, MO, USA).

### 4.2. Preparation of Rat Liver Mitochondria

Mitochondria were isolated from the liver of adult Wistar male rats using the differential centrifugation method. The liver was homogenized in an ice-cold isolation buffer containing 300 mM sucrose, 1 mM EGTA, and 10 mM HEPES–Tris (pH 7.4), and the homogenate was centrifuged at 600× *g* for 7 min at 4 °C. EGTA was added into the isolation medium to bind extracellular calcium ions. The supernatant fraction was centrifuged at 9000× *g* for 10 min. The pellet was collected and washed twice in the same medium (300 mM sucrose, 10 mM HEPES–Tris) but without EGTA. The final mitochondrial pellet was suspended in the washing medium to yield 60–80 mg of protein/mL.

### 4.3. Determination of Calcium Retention Capacity

The opening of the mPTP was induced by the sequential additions of CaCl_2_ to the incubation medium, as described earlier [42,43]. The mPTP opening was registered as a rapid rise in the concentration of calcium ions in the incubation medium. The concentration of Ca^2+^ was registered by a Ca^2+^-selective electrode (Nico, Moscow, Russia) in an open cuvette of volume 1 mL under continuous stirring. The mitochondrial calcium retention capacity (CRC) was defined as the total concentration of added Ca^2+^ required for pore opening. Mitochondria at a concentration of mitochondrial protein of 1–1.2 mg/mL were incubated in the buffer containing 125 mM KCl, 15 mM HEPES, 1.5 mM phosphate, and 5 mM succinate.

### 4.4. Assay of Swelling of Mitochondria

The swelling of mitochondria was measured at a wavelength of 540 nm using an Ocean Optics USB4000 spectrophotometer (Ocean Optic, Dunedin, FL, USA). Swelling was induced by the addition of CaCl_2_ and was assessed by changes in the optical density during incubation with compounds being tested. Mitochondria at a concentration of mitochondrial protein of 0.3–0.4 mg/mL were incubated in the buffer containing 125 mM KCl, 15 mM HEPES, 1.5 mM phosphate, and 5 mM succinate.

The effect of Tq on mPTP opening was also examined in the course of prolonged incubation with mitochondria in the presence of subthreshold concentrations of calcium ions and different, including physiological, concentrations of ATP. Under these conditions, mitochondrial swelling was determined by measuring a decrease in the optical density at 550 nm in a mitochondrial suspension using a plate reader (Infinite 200 Tecan, Groedig, Austria) and 96-well plates. The activation of the mPTP opening was characterized by the half-maximum swelling time (HMST), which reflects the time when the mPTP opening reaches half of the final amplitude, as described earlier [44,45]. Measurements were performed in KCl-based medium (120 mM KCl, 20 mM sucrose, 10 mM HEPES (pH 7.3), 2 mM MgCl_2_, and 2 mM KH_2_PO_4_) supplemented with 5 mM glutamate, 5 mM malate, and 10 µM EGTA. Other details are given in the figures and figure legends.

### 4.5. Determination of the Mitochondrial Membrane Potential

The difference in the electric potential on the inner mitochondrial membrane was measured from the redistribution of the lipophilic cation tetraphenylphosphonium (TPP^+^) between the incubation medium and mitochondria. The concentration of TPP^+^ in the incubation medium was recorded using a TPP^+^-selective electrode (Nico, Moscow, Russia).

### 4.6. Statistical Analysis

The data given represent the means (±) standard error of the means, S.D., of five to seven experiments or are the typical traces of three to five identical experiments with the use of different mitochondrial preparations. Statistical significance (*p* < 0.05) was determined using the Mann–Whitney U test and OriginPro 8 software.

## 5. Conclusions

The influence of Tq extends to mitochondria, specifically to the regulation of membrane permeability, promoting the activation of pore opening, probably through interaction with ANT, a component of the pore-forming complex. Tq actually decreased the resistance to the calcium load and eliminated the protective effect of ADP and ATP against the mPTP opening. The effect of Tq on the opening of mPTP is strongly dependent on the concentration of adenine nucleotides and, consequently, on the functional state of mitochondria in drug-resistant cells. Since chemoresistance may involve a variety of cellular protection mechanisms, including mitochondria-related ones, the direct influence of Tq on mitochondria can be considered as a subsidiary activity that promotes the sensitization of cells to different factors and stimuli. These data may be accounted for when using Tq in the therapy of drug-resistant pathologies as well as in the design of MDR inhibitors that are localized in plasma and mitochondrial membranes.

## Data Availability

The datasets generated during and/or analyzed during the current study are available from the corresponding author on reasonable request.

## Abbreviations

The following abbreviations are used in this manuscript:

ABC	ATP binding cassette
ANT	adenine nucleotide translocase
BA	bongkrekic acid
Catr	Carboxyatractylozide
CsA	cyclosporine A
HMST	half-maximum swelling time
mPTP	mitochondrial permeability transition pore
MDR	multidrug resistance
Tq	Tariquidar
TPP^+^	tetraphenylphosphonium ion
